# Co-creation of a step-by-step guide for specifying the test-management pathway to formulate focused guideline questions about healthcare related tests

**DOI:** 10.1186/s12874-024-02365-5

**Published:** 2024-10-16

**Authors:** Mariska K. Tuut, Gowri Gopalakrishna, Mariska M. Leeflang, Patrick M. Bossuyt, Trudy van der Weijden, Jako S. Burgers, Miranda W. Langendam

**Affiliations:** 1 Department of Family Medicine, Care and Public Health Research Institute CAPHRI, Maastricht University, PO Box 616, Maastricht, MD 6200 The Netherlands; 2 PROVA, Varsseveld, The Netherlands; 3 Department of Epidemiology, Maastricht University, Maastricht, The Netherlands; 4 Department Epidemiology and Data Science, Amsterdam UMC/University of Amsterdam, Amsterdam, The Netherlands; 5 Amsterdam Public Health Research Institute, Methodology Program, Amsterdam, The Netherlands; 6 Dutch College of General Practitioners, Utrecht, The Netherlands

**Keywords:** Healthcare related testing, Guidelines, Methodology, Education in guideline methods, Guidance

## Abstract

**Background:**

Guideline development on testing is known to be difficult for guideline developers. It requires consideration of various aspects, such as accuracy, purpose of testing, and consequences on management and people-important outcomes. This can be outlined in a test-management pathway. We aimed to create and user-test a step-by-step guide for guideline developers for designing a test-management pathway.

**Methods:**

Developmental design with a co-creative strategy. We created a draft step-by-step guide, that was user tested in a workshop with 19 experts, and by interviewing 7 guideline panel members.

**Results:**

Our proposed guide consists of five blocks of signalling questions: patients/population, index test(s), current practice/comparison/control, people-important outcomes, and the link between testing and outcome(s). The user testing led to refinement of the signalling questions, the use of inclusive terminology, and addition of a test-management pathway figure with detailed explanation.

**Conclusions:**

The step-by-step guide for formulating focused guideline questions regarding healthcare related testing can help in identifying relevant characteristics of the population, tests, and outcomes and to create a test management pathway. This should facilitate the formulation of evidence-based guideline recommendations about healthcare related testing.

**Supplementary Information:**

The online version contains supplementary material available at 10.1186/s12874-024-02365-5.

## Background

Guidelines aim to support decision making in healthcare practice [1]. The ultimate goal of a guideline is to improve or sustain health outcomes that are considered important from the perspective of the target population of the guidelines, so-called people-important outcomes, such as mortality and quality of life. A set of key questions define the scope of the guideline. Answers to such questions are based on systematic reviews of the evidence, combined with clinical expertise, and patients’ or consumers’ values and preferences. These are subsequently translated to guideline recommendations by the guideline panel [2]. The key questions include specific components, such as the population of interest, the intervention of interest and people-important outcomes [3].

In healthcare, tests can provide additional information about the past, current or future state of a person. The information may be relevant for diagnostic, prognostic, screening, monitoring, treatment (options), or other purposes. [4] Testing in itself usually has no direct effect on a patient/person’s health status. In addition, healthcare related tests are rarely used in isolation. They are typically included in a test-management pathway in which the information from testing is used to guide further actions [5–7].

The incremental value of test information will depend on population characteristics (e.g., features, symptoms, context and setting), test characteristics (e.g. sensitivity and specificity), management options following the test result and their downstream consequences on people-important outcomes [8]. The chain of these elements, starting from the testing process and ending in people-important outcomes is called the test-management pathway.

Different terms can be used for pathways that link testing to further clinical actions and people-important outcomes, such as test-treatment pathway, diagnostic pathway, clinical pathway, and analytical framework. In our study, we use the term ‘test-management pathway’ to be as inclusive as possible. Additionally, we prefer to use the term 'guidelines’, rather than ‘clinical practice guidelines’ so as to also include the public health domain. We also use the term ‘test’, ‘instead of ‘diagnostic test’, to include other purposes and settings of testing and test strategies [9]. Currently, the dominant source of evidence about testing most often comes from studies evaluating test performance, such as diagnostic accuracy [10]. Consequently, most guideline recommendations on testing are based on evidence concerning test accuracy only [11]. While having the best available estimates of a test’s clinical sensitivity and specificity is desirable, it is not sufficient for deciding whether testing should be recommended for use. Accuracy measures can help in estimating how many false positive and false negative results one may expect with testing, but this information should be put into context. For instance, the clinical performance of a test may differ in public health compared to a clinical setting due to factors such as the pre-test probability of the population being tested, previous tests conducted, and the resulting management decisions.

To develop recommendations about testing, guideline developers need to consider (a) the purpose of testing, (b) the desired downstream consequences of the test, in terms of minimal important changes in people-important outcomes, and (c) the link between test results, (healthcare) actions, and these outcomes [9, 12–14]. In addition, feasibility of the test (including sustainability), test burden (e.g., pain, time, discomfort), resources and costs need to be considered. The aim of testing is to improve people-important outcomes. A test-management pathway provides a visual representation of the essential steps required to move from testing to people-important outcomes, which is crucial in guideline development [15]. If guideline developers do not oversee and consider the consequences of testing, they cannot balance the relevant benefits and harms of testing. Relying on test accuracy solely may overestimate the added value of a test and may lead to overtesting, overdiagnosis and overtreatment.

Several agencies refer to the identification of test-management pathways in the evaluation of healthcare related tests and in drafting testing recommendations [13, 16–18]. These organisations mention the development of such pathways as part of the scoping process of a guideline, or as part of developing focused questions for systematic literature review. Studies in the guideline development community also support the integration of pathways in diagnostic test evaluation [19].

Identifying and outlining the elements of a test-management pathway in time and formulating focused questions about healthcare relating testing is not an easy task [20, 21]. Guideline developers have acknowledged that the inclusion of people-important outcomes in guideline development regarding testing is necessary but currently lacking. The formulation of key questions has been identified as a challenging aspect of this process, and there is consensus that education can play a crucial role in addressing this challenge [19]. Guideline developers therefore need support to formulate focused questions about testing at the start of a guideline development process.

Currently, a practical guide for the development of a test-management pathway is not available. Our group aimed to create, and user test a step-by-step guide on how to design such a test-management pathway aimed at guideline developers. The intention was that such a guide would assist guideline developers in formulating focused questions and evidence-based recommendations on testing.

## Methods

### General methodology

This project was based on a developmental design with a co-creative strategy. The initial creation of the step-by-step guide and the first phase of user testing were part of the DECIDE project, a 5-year project from January 2011 to December 2015, co-funded by the European Commission under the Seventh Framework Programme. Its objective was to build on the work of the GRADE Working Group to develop and evaluate methods for the dissemination of guidelines, including the evaluation of evidence and the development of recommendations about healthcare related tests [22]. Finalisation of the step-by-step guide and additional user testing was conducted in 2023. The authors are all researchers in the field of test evaluation and/or guideline development. They do not currently hold any active healthcare provider roles.

Firstly, the project team drafted a number of signalling questions per PICO element. Secondly, the step-by-step guide was co-created with two experts in the field and underwent user-testing with experts in the field and guideline panel members. This approach was selected to ensure comprehensive consideration of all relevant aspects. The Standards for Reporting Qualitative Research (SRQR) have been used to guide reporting of the research [23].

### Development of the step-by-step guide for creating a test-management pathway

The initial project team (GG, MML, PMB, MWL) selected the Population – Index test – Comparator – Outcome (PICO) elements as a starting point [24]. Using these elements and handbooks as basis (Agency for Healthcare Research and Quality, US Preventive Services Task Force, Cochrane handbook (for diagnostic test accuracy), GRADE for Diagnosis), the project team proposed a number of signalling questions for each PICO element, also based on their own expertise and experience in guideline development and study design [13, 16–18]. The aim of these questions was to facilitate guideline panel members in identifying issues that may need consideration when positioning the test of interest in its proposed pathway. The draft step-by-step guide was co-created in 2014 with one diagnostic test accuracy systematic reviewer and one guideline methodologist (MKT) within the network of the project team. With these experts, the test-management pathways for their topic of interest were drawn and their feedback was incorporated into the draft step-by-step guide.

### User testing workshop with experts

Workshop participants were (practising and non-practising) healthcare professionals and researchers with expertise and/or interest in guideline development who participated in the DECIDE Conference in Edinburgh in June 2014. We provided the participants with a 15-min introduction on the relevance of creating a test-management pathway in developing testing recommendations and presented our proposed approach. Then, test-management pathways were drafted using the step-by-step guide for two example questions: (1) B-type Natriuretic Peptide (BNP) testing for heart failure in elderly patients, and (2) CT-scanning in children with head injury who present at the emergency department. These topics were proposed by two volunteer participants. The test-management pathways were drafted through a collaborative effort between one researcher (PMB) and these volunteers in the presence of the other participants. Another researcher (MML) documented the process on a whiteboard. Two other project team members (GG and MWL) observed the process and took minutes.

Participants of the workshop gave input on these pathways, could ask questions and provided feedback. At the end of the workshop, participants completed a questionnaire about the usefulness and perceived challenges of the process used in the step-by-step guide (Appendix 1). The responses to these questionnaires were used to inform potential improvements to the step-by-step guide, including the wording of the steps.

### User testing with guideline panel members

In this phase, conducted in 2023, we used a before-after approach, in which we asked guideline panel members to formulate a guideline question on testing without and then with the use of the step-by-step guide. We selected a purposeful sample of at least five guideline panel members from an unspecified number of guideline panels, relying on our own network in the Netherlands. To be eligible, guideline panel members had to be involved at the start or in the development process of a guideline on testing. Guideline panel members were invited to participate per email. We provided the participants with a brief description of the project and planned two interviews with each participant to collect data.

The interviews were conducted by te first author of this study using the interview guides in Appendix 2. In the first interview conducted online, participants were asked to formulate a key question concerning the added value of a test for their guideline topic of interest. Then we sent our step-by-step guide, asked the participant to read this guide carefully and to note any questions, if the guide was not sufficiently clear. For this part of the study, we updated the step-by-step guide using inclusive terminology and translated it into Dutch (see Appendix 3).

In the second interview, conducted face-to-face, participants were asked to draw the test-management pathway for their test of interest using the step-by-step guide and answered any questions they had in the process. Then, participants were asked to adjust the originally formulated key question, if needed, and to provide feedback on the step-by-step guide and its use for this purpose.

All interviews were video recorded for notetaking and for incorporating feedback in the final version of the step-by-step guide.

## Results

### Development of the step-by-step guide for creating a test-management pathway

We created a guide consisting of five blocks of signalling questions concerning: (1) (P) patients/population, (2) (I) index test(s), (3) (C) current practice/comparison/control, (4) (O) people-important outcomes, and (5) link between testing and outcome(s). Pilot-testing of the draft step-by-step guide on diagnosis of eosinophilia in asthma and breast cancer screening resulted in refinement of the guide and the conclusion that the order in which the questions are addressed could vary, depending on the clinical question or topic. As an illustrative case, the pilot on breast cancer screening is reported in Appendix 4. The draft step-by-step guide is shown in Appendix 5.

### User testing with experts

Nineteen participants provided feedback on the step-by-step guide by completing the questionnaire (see Appendix 6 for detailed results). All agreed that drafting a test-management pathway is useful or even essential. Key issues raised were that more than one test-management pathway is likely for each guideline or key question and that all relevant stakeholders, such as healthcare professionals and consumers, should be involved in drafting the test-management pathway.

About half of the participants did not immediately see a direct link between the test-management pathway and derivation of relevant key questions. The participants who saw a link, valued the inclusion of people-important outcomes in the pathway and mentioned that making these outcomes explicit facilitates inferring changes in people-important outcomes when considering alternative testing in the test-management pathway.

Participants had different opinions about the ordering of the questions, the use of PICO, and the way the guidance was set up. People wondered why we chose a particular order in some cases (such as IPCO) and preferred sticking to the original PICO-order. One participant mentioned that setting should be explicitly included as an element in addition to the PICO. Some participants would have liked to see harms and patients’ values and preferences added to the outcome section as well. Following the user testing conducted in this phase of the study, no significant amendments were made to the step-by-step guide. However, a number of refinements have been incorporated.

All participants, except one, would consider using the test-management pathway in their guideline work if step-by-step user guidance would be available. About half of the participants preferred an open question format for the guide, while others favored a checklist format. One participant suggested producing software that could help in the visualization of the pathway.

Besides knowledge about tests, diagnostic research, and evidence-based medicine, participants indicated that they would value training in interviewing skills and in moderating discussions involving the guideline panel. This training could have different formats, such as video tutorials, hands-on practicing, online training, and/or a more detailed step-by-step checklist.

### User testing with guideline panel members

During the final round of user testing, seven guideline panel members from two Dutch panels on the topics secondary care for people with autoimmune hemolytic anemia and primary care for women with dysmenorrhea were included. The participants included two clinical chemists, one hematologist, one general practitioner, and three patient representatives. In the first online interview, all interviewees were able to formulate an initial testing question. Prior to the second interview, six participants had reviewed the step-by-step user guide that was provided after the initial interview. During the second interview, all participants were able to create a test-management pathway for their question of interest, by using the step-by-step guide and instructions provided by the interviewer.

After drafting the test-management pathway for their test of interest, six participants adjusted their original question. These adjustments included:

- Refining the population of interest (such as adding information about the setting and earlier tests performed).
- Specifying the purpose of the test and its place in the test-management pathway.
- Addressing practical aspects of testing, such as difficulties in performing the test adequately.
- Defining test burden.
- Adding the impact of testing in terms of impact on people-important outcomes.

Participants found the step-by-step guide helpful for structuring questions and defining the purpose and impact of the test of interest. They also found the examples provided useful and intended to use the guide in a guideline panel setting. Suggestions for improvement included the need for instruction for usage, a figure/example of a test-management pathway, and the explanation of terminology for patient representatives.

### Final step-by-step guide

In the final version of the step-by-step guide, we added an introduction, instructions, and a figure with the test-management pathway. The final version of the step-by-step guide is presented in Table 1.

**Table 1:**
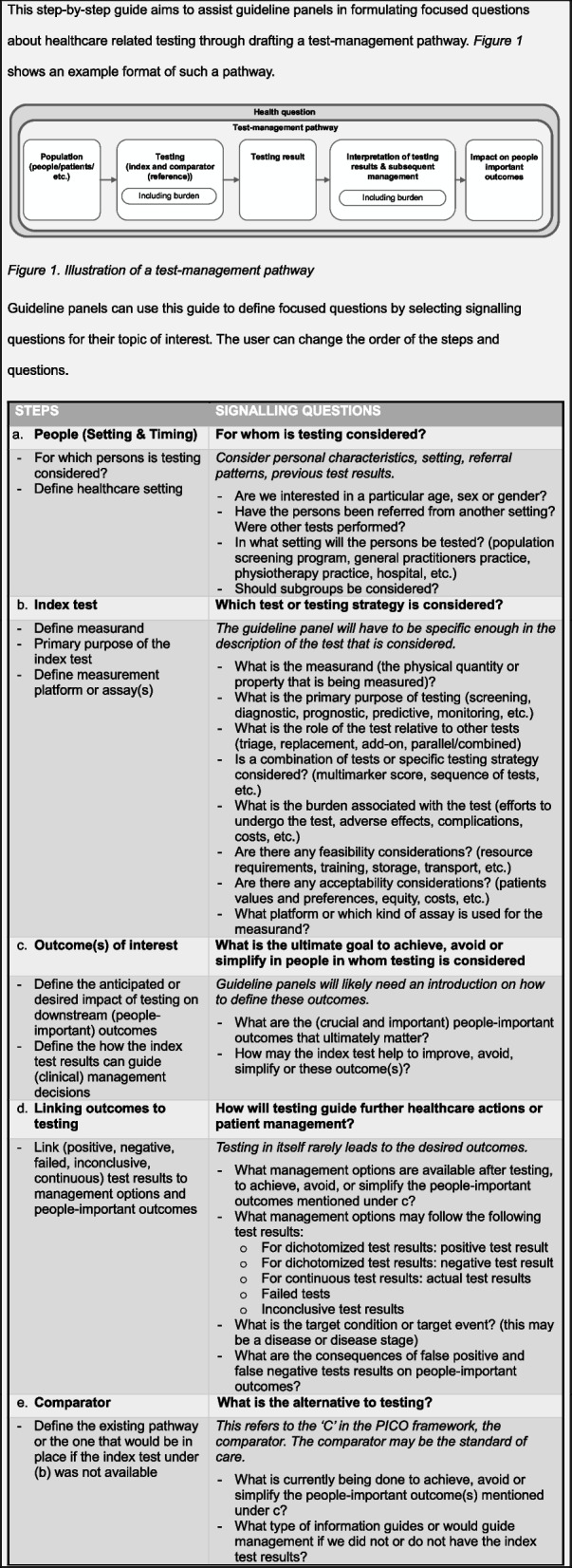
Final step-by-step guide for developing a test-management pathway

## Discussion

This study presents a step-by-step guide for guideline panels to formulate focused questions regarding healthcare related testing. The guide can aid in creating a test-management pathway by identifying relevant characteristics of the population, tests, and outcomes of interest when developing clinical practice guidelines or public health guidelines.

The formulation of focused rather than broad questions allows explicit consideration of factors beyond test accuracy. These include feasibility, timing, test burden, management effectiveness and impact on people-important outcomes. Furthermore, the step-by-step guide offers the possibility of distinguishing between different patient subgroups. It is assumed that this approach will result in recommendations that are better balanced and that are explicitly aimed at improving people-important outcomes. This may lead to less overtesting, overdiagnosis and subsequent overtreatment, which would be beneficial from a patient’s perspective as well as from a public health perspective.

Explicit step-by-step guidance on how to actually derive such pathways is limited in the existing guidance. So far, we have not been able to identify studies reporting on the experience of users applying these approaches. Test-management pathways and concepts have been presented earlier as a tool for setting the scene and framing the question(s) in a guideline development or test accuracy review process [13, 16–18]. The AHRQ and the USPSTF refer to the development of such pathways as a guide to help in formulating specific key questions [16, 17]. Both organizations use the term ‘Analytical Framework’, which they use both for intervention related questions and for test-related questions as a way of going from a more ambiguous initial claim to a specific answerable guideline or review question. The Cochrane Handbook for Systematic Reviews of Diagnostic Test Accuracy uses the term ‘Clinical pathway’ to outline how patients might present, when they would be considered for testing and the role of the test [18].

### Limitations of this study

The first user testing was done with participants experienced in test research and/or guideline development. Most participants in these sessions had prior knowledge about evaluation of tests and development of guideline recommendations about tests. It is therefore questionable whether the results of these user testing phases are applicable to guideline panels with less methodological expertise and experience. It is known that guideline panels are quite familiar with treatment guidelines and have limited initial understanding of the link between testing, downstream management, and people-important outcomes [19]. Thus, more guidance might be needed. We therefore also user tested the step-by-step guide with guideline panel members.

We tested the step-by-step guide in a limited number of persons and panels. They were recruited as a purposeful sample of experienced and less experienced guideline developers and reviewers with varying expertise and experience in test research. During user testing with guideline panel members, we observed that participants considered reformulating their initial test question after using the step-by-step guide. The instructions on creating questions for a guideline reflect a natural situation, as the development of guideline questions typically involves a group process led by a guideline methodologist.

Some data were collected almost a decade ago. Therefore, we adjusted the step-by step guide using terminology that is inclusive and more widely accepted by today’s standards. We believe that our findings are still relevant after adjustment and by adding a test-management pathway visualization and instructions for use.

The primary focus for our step-by-step guide is to raise awareness on people-important outcomes. Recommendations on tests can be focused on other aims as well, such as simplifying or streamlining the healthcare process, to reduce costs, to increase efficiency, or to reduce painful procedures. We agree that these considerations matter but in all cases the people-important outcomes should also be considered.

### Implications for practice

The step-by-step guide is meant to be used in a flexible manner. During the user testing sessions, there was some debate about where to start in the process: with the ‘P’ for people or population, or with the ‘I’ for index test. We think this may depend on the overall question to be answered. For example, if an index test is central in the question, such as ‘Should we use this test in these patients?’, then starting with the ‘I’ seems to result in a more focused process. On the other hand, if the question is about whether a test should be recommended in a particular setting, then first describing the ‘P’ and setting may be more helpful.

We suggest utilizing the step-by-step guide in the guideline panel process during the stage of (PICO) question generation [25, 26]. Drafting a test-management pathway will often be an iterative process. Further modifications of the pathway during guideline development may be needed. Our step-by-step guide can help in this process. Although using the step-by-step guide in the guideline development process may require some time, it is anticipated that this will facilitate the specification of more focused questions. We expect that this might reduce the time required at a later stage in the guideline development process and will enable the development of targeted and more balanced recommendations.

Though our focus was on guideline development, we have experienced that the user guide can also be useful in other areas of decision making. As authors, we have used it in developing recommendations about coverage in a healthcare benefits package. We have also used it when designing clinical trials and deciding on the proper performance measures. Within the recently introduced European Union In Vitro Diagnostics Regulation, clinical performance should be informative about the clinical utility of the test, reflecting the purpose of testing in the intended use setting and population.

Users expressed that a digital tool that is both intuitive and flexible would be helpful for drawing test-management pathways, and to document the iterations it goes through. We suggest developing an online tool, for example as a feature in software such as RevMan and/or GRADEPro.

### Implications for research

In developing a test-management pathway we encourage further evaluations of the step-by-step guide in guideline panels. This could result in additional tools and instruments to facilitate the development of recommendations about tests and testing.

Once the pathway is defined, research evidence to support assumptions made in the pathway can be sought. One could also use the test-management pathway to decide on minimally acceptable performance of the tests, and to evaluate limitations in the applicability of research findings.

## Conclusion

We have developed a step-by-step guide, for guideline developers, to create a test-management pathway, which can be helpful in formulating focused questions regarding healthcare related testing. The guide facilitates guideline developers in defining structured questions by identifying relevant characteristics of the population, tests, and outcomes of interest. This is an essential step in the development of informed, evidence-based, guideline recommendations for healthcare related testing.

## Supplementary Information

Supplementary Material 1.

Supplementary Material 2.

Supplementary Material 3.

Supplementary Material 4.

Supplementary Material 5.

Supplementary Material 6.

## Data Availability

The datasets used and/or analysed during the current study are available from the corresponding author on reasonable request.

## Abbreviations

AHRQ: Agency for Healthcare Research and Quality
BNP: B-type Natriuretic Peptide
CT: Computed Tomography
DECIDE: Developing and Evaluating Communication strategies to support Informed Decisions and practice based on Evidence
GRADE: Grading of Recommendations Assessment, Development and Evaluation
IPCO: Index test - Population - Comparator - Outcome
PICO: Population - Index Test - Comparator - Outcome
RevMan: Review Manager
SRQR: Standards for Reporting Qualitative Research
USPSTF: US Preventive Services Task Force
