# Supplementary material for: Co-creation of a step-by-step guide for specifying the test-management pathway to formulate focused guideline questions about healthcare related tests
Source: BMC Med Res Methodol. 2024 Oct 16;24:241. doi: 10.1186/s12874-024-02365-5 (PMC11481243; doi:10.1186/s12874-024-02365-5)
Supplement: Supplementary file 1 — Supplementary Material 1. [file 12874_2024_2365_MOESM1_ESM.docx]

# Appendix 1: Questionnaire used to get feedback at DECIDE the user testing workshop

| 1a. Do you think the approach we presented today can be useful in understanding the context and place of a test? |
| --- |
| 1b. Does it give insight into the different types of evidence needed? |
| 2. What do you think of the structure (PICO) and flow of questions in this demo? |
| 3. Would you consider using this approach in developing a guideline about diagnostic tests? |
| 4. What level training would you need to successfully define such a pathway for a guideline? |
| 5. What kind of training would you prefer?   - Hands-on workshop - Online training / webinar - Structured guidance (step-by-step user guide) - Video-taped examples - Other, please specify |
| 6. Would you prefer an open (semi structured interview) or structured (checklist) approach? |
| 7. Do you have any suggestions for improvement? |
