# Supplementary material for: Co-creation of a step-by-step guide for specifying the test-management pathway to formulate focused guideline questions about healthcare related tests
Source: BMC Med Res Methodol. 2024 Oct 16;24:241. doi: 10.1186/s12874-024-02365-5 (PMC11481243; doi:10.1186/s12874-024-02365-5)
Supplement: Supplementary file 2 — Supplementary Material 2. [file 12874_2024_2365_MOESM2_ESM.docx]

# Appendix 2. Interview guides for the user testing sessions with guideline panel members

[translated from Dutch]

**First interview (online)**

- Intro
- Explanation of the context and purpose of the study (guideline development on healthcare related testing, study to test a step-by-step guide aimed at facilitating the formulation of key questions)
- START VIDEO RECORDING
- Would you please formulate a key question on the use of one or more tests for the guideline that you are involved in?
- Thank participant
- STOP TAPE RECORDING
- Explanation of the remainder of the study: you will receive the step-by-step guide by email with a request to read it critically and note questions/clarifications
- Schedule appointment for second (on site) interview
- Thank participant again and close session

**Second interview (on site)**

- START VIDEO RECORDING
- Repeat key question formulated in the first (online) interview
- Concerning the step-by-step guide:
  - Did you manage to read through/study the step-by-step guide?
  - Do you have any preliminary questions/comments regarding the step-by-step guide?
- Going through the manual (participant in the lead, interviewer can adjust and possibly provide clarification):
  - Complete test-management pathway for the same testing situation
  - Possibly reformulate initial key question
  - Determine whether and in what way the key question has been changed
- Ask for feedback on the step-by-step guide
- STOP VIDEO RECORDING
