# Supplementary material for: Co-creation of a step-by-step guide for specifying the test-management pathway to formulate focused guideline questions about healthcare related tests
Source: BMC Med Res Methodol. 2024 Oct 16;24:241. doi: 10.1186/s12874-024-02365-5 (PMC11481243; doi:10.1186/s12874-024-02365-5)
Supplement: Supplementary file 3 — Supplementary Material 3. [file 12874_2024_2365_MOESM3_ESM.docx]

# Appendix 3. Updated step-by-step guide for developing a test-management pathway

| STEPS | SIGNALLING QUESTIONS |
| --- | --- |
| 1. People (Setting & Timing) | **In whom is testing considered?** |
| - Define the eligibility criteria: in which persons is testing considered? - Define healthcare setting | *Consider personal characteristics, setting, referral patterns, previous test results.*   - Are we interested in a particular age, sex or gender? - Have the persons been referred from another setting? Were other tests performed? - In what setting will the persons be tested? (population screening program, general practitioners practice, physiotherapy practice, hospital, etc.) - Should subgroups be considered? |
| 1. Index test | **Which test or testing strategy is considered?** |
| - Define measurand - Primary purpose of the index test - Define measurement platform or assay(s) | *The guideline panel will have to be specific enough in the description of the test that is considered.*   - What is the measurand (the physical quantity or property that is being measured)? - What is the primary purpose of testing (screening, diagnostic, prognostic, predictive, monitoring, etc.) - What is the role of the test relative to other tests (triage, replacement, add-on, parallel/combined) - Is a combination of tests or specific testing strategy considered? (multimarker score, sequence of tests, etc.) - What is the burden associated with the test (efforts to undergo the test, adverse effects, complications, costs, etc.) - Are there any feasibility considerations? (resource requirements, training, storage, transport, etc.) - Are there any acceptability considerations? (patients values and preferences, equity, costs, etc.) - What platform or which kind of assay is used for the measurand? |
| 1. Outcome(s) of interest | **What is the ultimate goal to achieve, avoid or simplify in people in whom testing is considered** |
| - Define the anticipated or desired impact of testing on downstream (people-important) outcomes - Define the how the index test results can guide (clinical) management decisions | *Guideline panels will likely need an introduction on how to define these outcomes.*   - What are the (crucial and important) people-important outcomes that ultimately matter? - How may the index test help to improve, avoid, simplify or these outcome(s)? |
| 1. Linking outcomes to testing | **How will testing guide further healthcare actions or patient management?** |
| - Link (positive, negative, failed, inconclusive, continuous) test results to management options and people-important outcomes | *Testing in itself rarely leads to the desired outcomes.*   - What management options are available after testing, to achieve, avoid, or simplify the people-important outcomes mentioned under c? - What management options may follow the following test results:   - For dichotomized test results: positive test result   - For dichotomized test results: negative test result   - For continuous test results: actual test results   - Failed tests   - Inconclusive test results - What is the target condition or target event? (this may be a disease or disease stage) - What are the consequences of false positive and false negative tests results on people-important outcomes? |
| 1. Comparator | **What is the alternative to testing?** |
| - Define the existing pathway or the one that would be in place if the index test under (b) was not available | *This refers to the ‘C’ in the PICO framework, the comparator. The comparator may be the standard of care.*   - What is currently being done to achieve, avoid or simplify the people-important outcome(s) mentioned under c? - What type of information guides or would guide management if we did not or do not have the index test results? |

## Translation in Dutch

| STAPPEN | VRAGEN |
| --- | --- |
| 1. Populatie (Setting & Timing) (P) | **Bij wie wordt testen overwogen?** |
| - Beschrijf de in- en exclusiecriteria: bij welke personen wordt testen overwogen? - Beschrijf de gezondheidszorg setting | *Overweeg persoonskenmerken, setting, verwijzingen, voorgaande testresultaten*   - Zijn we geïnteresseerd in een bepaalde leeftijd, geslacht of sekse? - Zijn de personen doorverwezen vanuit een andere omgeving? Zijn er andere tests uitgevoerd? - In welke setting worden de personen getest? (bevolkingsonderzoek, huisartsenpraktijk, fysiotherapiepraktijk, ziekenhuis, etc.) - Moeten subgroepen worden overwogen? |
| 1. Indextest (I) | **Welke test of teststrategie wordt overwogen?** |
| - Definieer meetgrootheid - Primair doel van de indextest - Leg meetsysteem of assay(s) vast | *De richtlijnwerkgroep moet specifiek genoeg zijn in de beschrijving van de test die overwogen wordt*   - Wat is de te meten grootheid (de fysieke grootheid of eigenschap die gemeten wordt)? - Wat is het primaire doel van de test (screening, diagnostisch, prognostisch, voorspellend, monitoring, etc.) - Wat is de rol van de test ten opzichte van andere tests (triage, vervanging, aanvulling, parallel/gecombineerd)? - Wordt een combinatie van tests of een specifieke teststrategie overwogen? (multimarker score, volgorde van testen, etc.) - Wat is de belasting van de test (moeite om de test te ondergaan, bijwerkingen, complicaties, kosten, enz.) - Zijn er implementatieknelpunten? (benodigde middelen, training, opslag, transport, etc.) - Zijn er knelpunten met betrekking tot aanvaardbaarheid? (waarden en voorkeuren van patiënten, rechtvaardigheid, kosten, etc.) - Welk platform of welk soort assay wordt gebruikt voor de te meten grootheid? |
| 1. Uitkomsten (O) | **Wat is het uiteindelijke doel om te bereiken, vermijden of vereenvoudigen bij mensen bij wie testen wordt overwogen?** |
| - Beschrijf de verwachte of gewenst impact van testen of patiëntrelevante uitkomstmaten - Beschrijf hoe de resultaten van de indextest (klinisch) beleid kunnen bepalen | *Richtlijnwerkgroepen hebben mogelijk een uitleg nodig over het bepalen van patiëntrelevante uitkomstmaten*   - Wat zijn de (cruciale en belangrijke) patiëntrelevante uitkomstmaten die uiteindelijk van belang zijn? - Hoe kan de indextest helpen om deze uitkomst(en) te verbeteren, vermijden of vereenvoudigen? |
| 1. Koppelen van uitkomsten aan testen | **Hoe kan het testen van invloed zijn op het beleid bij de patiënt?** |
| - Koppel testresultaten (positief, negatief, mislukt, inconclusief, continu) aan beleid en patiëntrelevante uitkomstmaten | *Testen zelf leidt zelden tot de gewenste uitkomsten.*   - Welke beleidsopties zijn beschikbaar na testen, om de onder c genoemde patiëntrelevante uitkomstmaten te verbeteren, vermijden of vereenvoudigen? - Welke beleidsopties kunnen volgen op de volgende testresultaten:   - Voor dichotome testresultaten: positief testresultaat   - Voor dichotome testresultaten: negatief testresultaat   - Voor continue testresultaten: actuele testresultaten   - Mislukte testen   - Inconclusieve testen - Wat is de beoogde conditie of gebeurtenis waarop de test is gericht (dit kan bijv. een ziekte of stadium zijn) - Wat zijn de gevolgen van fout-positieve en fout-negatieve testresultaten op patiëntrelevante uitkomstmaten? |
| 1. Vergelijking (C) | **Wat is het alternatief voor testen?** |
| - Beschrijf de bestaande test-management strategie of de test-management strategie die van toepassing zou zijn als de indextest onder b) niet beschikbaar zou zijn. | *Dit verwijst naar de C in de PICO, de controle/vergelijking. Dit kan standaardzorg zijn.*   - Wat is de huidige test-management strategie om patiëntrelevante uitkomsten (genoemd bij c) te bereiken, vermijden of vereenvoudigen? - Op basis van welke informatie wordt het beleid (of zou het worden) bepaald als de indextest niet beschikbaar zou zijn? |
