# Supplementary material for: Co-creation of a step-by-step guide for specifying the test-management pathway to formulate focused guideline questions about healthcare related tests
Source: BMC Med Res Methodol. 2024 Oct 16;24:241. doi: 10.1186/s12874-024-02365-5 (PMC11481243; doi:10.1186/s12874-024-02365-5)
Supplement: Supplementary file 4 — Supplementary Material 4. [file 12874_2024_2365_MOESM4_ESM.docx]

# Appendix 4. Illustrative case of pilot testing

*Supplementary table 1* describes how the initially broad key question brought in the pilot was clarified by the interview process leading to the identification of specific issues to be clarified. These were included as part of the test-management pathway (*Supplementary figure 1*) that were originally not explicitly identified by the user prior to applying this approach.

**Supplementary table 1. Example of an initially ambiguous key question clarified through test-management pathway development**

| ***General topic*** | MRI to replace mammography in breast cancer screening |
| --- | --- |
| ***Initially ambiguous non-specific / broad key question*** | Will using MRI instead of mammography to screen for breast cancer lead to a higher survival of women at high risk of developing breast cancer? |
| ***Key concerns identified through the step-by-step approach of pathway development*** | |
| Patients, Setting, Timing (P) | Naïve, high-risk women (i.e. with a family history of breast cancer) below the age of 50 years, identified through general practitioners and then referred to secondary care where they will enter the pathway |
| Index test(s) (I) | MRI is the replacement test being considered, although not all hospitals will have this facility |
| Comparison or Existing test/strategy (C) | Mammography is the existing test/strategy and the available treatment options identified were combinations of radiotherapy/chemotherapy/ surgery/immunotherapy depending on tumor type and stage with varying prognosis |
| Outcomes of interest (O) | - Increased (breast-cancer specific / disease-free) survival among high-risk women through early detection - Reduced anxiety of disease and hence improve quality of life through reassurance - Impact on clinical management decisions would involve re-testing after a year for true negatives and false negatives; follow up in six months for false positives |
| Linking outcomes to test accuracy | - True positives: will go through biopsy and receive treatment depending on risk category - True negatives: will be re-tested a year later - False positives: unnecessary biopsy and anxiety; will be monitored in approximately 6 months’ time - False negatives: wrongly reassured and may only be diagnosed a year later |

**
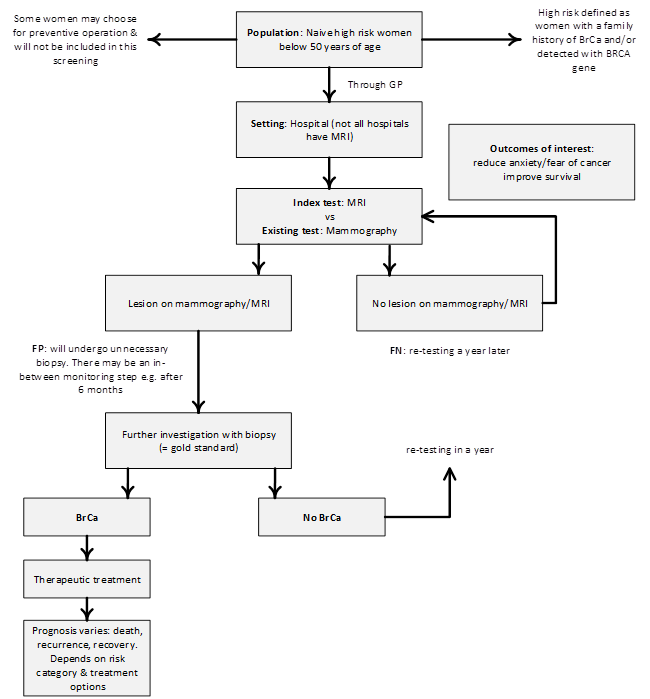
**

**Supplementary figure 1. Illustrative example of a test-management pathway: MRI in women at high risk of breast cancer**

In this example, MRI (the index test) is more sensitive than mammography (the comparator), but it is unclear whether it also leads to a better survival in this patient group. Randomized controlled studies exploring the differences between MRI and mammography on people-important outcomes such as mortality are lacking. The test-management pathway will generally be the same for both tests. Any difference in survival between the two groups (MRI versus mammography) will therefore be mainly driven by differences in test performance (such as sensitivity and specificity) in results between the two tests. However, a more accurate test does not necessarily lead to a survival benefit. For example, women at high risk of developing breast cancer, because of hereditary factors, may develop a more aggressive tumor leading to a higher mortality, independent of the stage in which it is diagnosed. In that case, screening may not improve survival and a test with a higher accuracy may not have any net health benefit above a test with a lower accuracy.
