# Supplementary material for: Co-creation of a step-by-step guide for specifying the test-management pathway to formulate focused guideline questions about healthcare related tests
Source: BMC Med Res Methodol. 2024 Oct 16;24:241. doi: 10.1186/s12874-024-02365-5 (PMC11481243; doi:10.1186/s12874-024-02365-5)
Supplement: Supplementary file 5 — Supplementary Material 5. [file 12874_2024_2365_MOESM5_ESM.docx]

# Appendix 5. Draft step-by-step guide for developing a test-management pathway

| **Steps** | **Trigger questions** |
| --- | --- |
| 1. **Patients (Setting & Timing)** |  |
| - Define patient characteristics - Define the target condition - Define prior tests & setting | **What kind of patients are being considered?**  *Consider patient characteristics, setting, referral patterns*  **Trigger questions:**   - Are you interested in a particular age, gender, etc? - What is the disease or disease stage that the index test is intended to identify? - Have the patients been referred from another place and have other tests been done there? - In what situation will the patients be tested? Is it a screening situation, or at the GP’s, or somewhere else? - What is the healthcare setting in which the index test will be applied: community, primary, or secondary care? |
| 1. **Index test** |  |
| - Role & purpose of the index test - Point in the pathway where the index test might be considered - Test variations, if relevant - Test specifications | **What is the test or tests of interest?**  *The guideline development group must have a clear idea of where the new test may be placed in the pathway. There may be a need to go back to this item once the pathway is better defined*  **Trigger questions:**   - What is the purpose of the test i.e. diagnostic, prognostic, monitoring etc - What is the role of a test (i.e. triage, replacement, add-on in comparison to existing test(s)) - What are the test variations i.e. are there different manufacturers of a test, who will be operating and interpreting the test, and is there more than one threshold to be considered - What are the test specifications to need to be considered (i.e resource requirements, training, translations, specialized equipment or conditions etc) |
| 1. **Comparison or Existing test(s)/strategy** |  |
| - Define the pathway that would be in place if the index test was not available. | **What is the comparison or existing test strategy to avoid/achieve the outcome of interest?**  *This refers to the ‘C’ in the PICO framework, the comparator. The comparator may be standard care.*  **Trigger questions:**   - What is currently being done to avoid/achieve outcome(s)? - What would we do if we do not use the index test? - What will alternatively guide clinical decision-making? - What treatment options are available? |
| 1. **Outcome(s) of interest to avoid or achieve** |  |
| - Define the impact of the index test on downstream (patient) outcomes - Define the impact of the index test on clinical management decisions | **What are we trying to avoid, achieve or simplify in patients?**  *Guideline development groups will likely need an introduction on how to define patient outcomes, and its link to medical testing. There may be different outcomes for different settings.*  **Trigger questions:**   - How may the introduction of the index test help to avoid, simplify or improve these (patient) outcome(s)? - What is the potential impact of the index test on clinical management decisions e.g. decisions involving referral for further investigation or treatment options etc. |
| 1. **Linking outcomes to the testing** |  |
| - What actions follow after the different test results? - How do these actions impact the four test accuracy categories (TP, TN,FP, FN) and inconclusive test results? - If possible, one could provide weights to different downstream outcomes | **Trigger questions:**  **For true positives (TP) and false positives (FP):**   - Will patients with a positive test be: referred to a specialist, referred for subsequent testing, treated for the condition? - What should ideally be done in those with the target condition? - Is there effective treatment available? - What will be possible outcomes for patients who do not have the target condition and test positive?   **For false negatives (FN) and true negative (TN):**   - What will happen to those patients who test negative? - Is it assumed that these patients are ‘healthy’ or will they probably have another disease than the target condition? - Will the patients be re-tested in due time? - How likely is it that this condition will be missed, or will there be a delayed diagnosis in the false negatives? - What is the prognosis of patients with the target condition if treatment is being withheld?   **For inconclusive test results:**   - What will happen to these patients, will they be re-tested and within what duration and the number of times |
