# Supplementary material for: Co-creation of a step-by-step guide for specifying the test-management pathway to formulate focused guideline questions about healthcare related tests
Source: BMC Med Res Methodol. 2024 Oct 16;24:241. doi: 10.1186/s12874-024-02365-5 (PMC11481243; doi:10.1186/s12874-024-02365-5)
Supplement: Supplementary file 6 — Supplementary Material 6. [file 12874_2024_2365_MOESM6_ESM.docx]

# Appendix 6. Detailed feedback on the step-by-step guide from DECIDE workshop participants

| **Question**  (agreement) | **Agreement** | **Feedback** |
| --- | --- | --- |
| **Do you think the approach we presented today is useful in understanding the context and place of a test? (yes/no)** | 19/19 | - The pathway definition does not necessarily need to be the first step in guideline development - There may be more than one pathway for a given test strategy - Some pathways maybe challenging due to variation in practice - A multidisciplinary team may be needed going across primary, secondary, and tertiary care in order to establish accurate, comprehensive, factual information on the pathway |
| **Does it give insight into the different types of evidence needed? (yes/no)** | 14/17 | More information and guidance on defining clear inclusion and exclusion criteria for the evidence search is needed |
| **What do you think of the structure (PICO) and flow of questions in this approach?** |  | - The visualization was helpful to get a picture of missing information - The approach is good as an initial starting point to developing the pathway - It is difficult to establish a smooth flow of questioning since discussions tended to go off tangent into individual benefits versus population benefits and/or harms or if an entire panel is involved - There was expectation of a software to support this approach - Starting and ending with the patient outcomes maybe more appropriate - The Interviewer must have some background information about the health problem - The approach should be more about Bayesian steps of pre-test probability estimates derivation and sequence of diagnostic ins- and rule-outs. - Defining the clinical outcomes was difficult |
| **Would you consider using this approach in developing a guideline? (yes/no)** | 18/19 | - The process should engage the full guideline panel - Different strategies could be used for getting different perspectives: e.g. focus group or one to one interviewing with an inductive approach - These pathways are not only useful for diagnostic tests but also for treatments - It seems time-consuming with a need for healthcare professionals and guideline panels to be "trained" or "used to" this approach - The approach was not explicit enough on how it can be used in practice to help guideline development / make recommendation - Discussions about the reference standard can cause discussions to go off tangent and to reach consensus - Starting with defining the patients and not the index test might be more logical |
| **What level of training/support would you need to successfully define such a pathway for a guideline?** |  | - Basic understanding of diagnostic test research - Search strategies and selection criteria for studies - Flow of the questions: how to select the most important questions to keep the workload feasible for the review/guideline group - How to best visualize the final output and capture the iterations in between Interviewing and group facilitation skills - Knowledge in Evidence Based Medicine and guideline development - Hands-on one-to-one training or another ‘live demonstration’ |
| **What kind of training would you prefer?** |  | - Hands-on workshop (n=11) - Structured guidance (step-by-step guide) (n=10) - Online training / webinar (n=4) - Video-taped examples (n=4) - Other (journal series, workshops like this, real examples) (n=3) |
| **Would you prefer an open, semi structured or structured (checklist) approach?** |  | - Structured approach (n=3) - Semi-structured interview as so many variations and it allows for clarifications (n=9) - Both could be helpful, so that you could choose, depending on the topic and issues addressed, structured approach as starting point with most important questions plus additional questions for anything else at the interview) (n=4) - Unsure (n=1) - No reply (n=2) |
| **Do you have any suggestions for improvement?** |  | - Possible harmful effects (that are not foreseen in the initial hypothesis/key question) deserve more attention - Give multiple examples of pathways and how to visualize them effectively - Illustrate pros ,cons and practicalities of using this approach using real examples - More focus on contextualizing the question |
